# A quantitative study of methanol/sorbitol co-feeding process of a *Pichia pastoris* Mut^+^/pAOX1-lacZ strain

**DOI:** 10.1186/1475-2859-12-33

**Published:** 2013-04-08

**Authors:** Hongxing Niu, Laurent Jost, Nathalie Pirlot, Hosni Sassi, Marc Daukandt, Christian Rodriguez, Patrick Fickers

**Affiliations:** 1 Unité de Biotechnologies et Bioprocédés, Université libre de Bruxelles, Av. F.-D. Roosevelt 50 CP 165/61, 1050 Brussels, Belgium; 2 Eurogentec S.A., Rue du Bois Saint Jean, n°14, 4102 Seraing, Belgium

**Keywords:** *Pichia pastoris*, pAOX1, β-galactosidase, Methanol/sorbitol co-feeding, Metabolic flux analysis, Transient continuous culture, Fed batch culture

## Abstract

**Background:**

One of the main challenges for heterologous protein production by the methylotrophic yeast *Pichia pastoris* at large-scale is related to its high oxygen demand. A promising solution is a co-feeding strategy based on a methanol/sorbitol mixture during the induction phase. Nonetheless, a deep understanding of the cellular physiology and the regulation of the AOX1 promoter, used to govern heterologous protein production, during this co-feeding strategy is still scarce.

**Results:**

Transient continuous cultures with a dilution rate of 0.023 h^-1^ at 25°C were performed to quantitatively assess the benefits of a methanol/sorbitol co-feeding process with a Mut^+^ strain in which the pAOX1-lacZ construct served as a reporter gene. Cell growth and metabolism, including O_2_ consumption together with CO_2_ and heat production were analyzed with regard to a linear change of methanol fraction in the mixed feeding media. In addition, the regulation of the promoter AOX1 was investigated by means of β-galactosidase measurements. Our results demonstrated that the cell-specific oxygen consumption (qO_2_) could be reduced by decreasing the methanol fraction in the feeding media. More interestingly, maximal β-galactosidase cell-specific activity (>7500 Miller unit) and thus, optimal pAOX1 induction, was achieved and maintained in the range of 0.45 ~ 0.75 C-mol/C-mol of methanol fraction. In addition, the qO_2_ was reduced by 30% at most in those conditions. Based on a simplified metabolic network, metabolic flux analysis (MFA) was performed to quantify intracellular metabolic flux distributions during the transient continuous cultures, which further shed light on the advantages of methanol/sorbitol co-feeding process. Finally, our observations were further validated in fed-batch cultures.

**Conclusion:**

This study brings quantitative insight into the co-feeding process, which provides valuable data for the control of methanol/sorbitol co-feeding, aiming at enhancing biomass and heterologous protein productivities under given oxygen supply. According to our results, β-galactosidase productivity could be improved about 40% using the optimally mixed feed.

## Background

The methylotrophic yeast *Pichia pastoris* has become one of the most frequently used expression systems for heterologous proteins [1-3] with many advantages over *Saccharomyces cerevisiae*, such as availability of engineered strains capable of humanized glycosylation [4,5], better protein secretion efficiency, ability to grow on defined media with high biomass yield and existence of promoter, such as pAOX1, tightly regulated by methanol. However, since methanol is a high-degree reductant with high heat of combustion [6,7], it leads to one major challenge during methanol induction phase at large-scale. Because heat production is almost linearly correlated with oxygen consumption in aerobic culture [8], the challenge is how to reduce oxygen consumption (accordingly, pO_2_ is controllable) without affecting protein productivity?

One possible answer is to use co-substrates, such as glycerol [9-11], glucose [12,13] or sorbitol [14-19] to partially replace or at least complement methanol during protein production phase. The most promising co-substrate is sorbitol because it is a low-degree reductant and a non-repressing carbon source for pAOX1 [14]. The benefits of mixed feed of sorbitol and methanol have been widely characterized with the aims of reducing oxygen consumption without loss of protein productivity. However, very few studies have been performed to quantitatively analyze the cellular physiology during the co-feeding [20,21]; focusing only on the final product (i.e. a secreted protein) without any insights on the pAOX1 regulation.

In this study, we report on the quantitative characterization of *P. pastoris* cell metabolism, with special emphasis on the quantification of pAOX1 induction during a methanol/sorbitol co-feeding process by means of transient continuous cultures [7,22] and metabolic flux analysis. In addition, our experimental findings were further confirmed in fed-batch cultures.

## Results and discussion

### Strain characterization with transient continuous cultures

Preliminary experiments demonstrated that maximum specific growth rates on methanol and sorbitol based media were equal to 0.10 ± 0.02 h^-1^ and 0.04 ± 0.01 h^-1^, respectively (data not shown). Thus, to avoid washout of biomass, the methanol/sorbitol co-feeding process was examined at a constant dilution rate of 0.023 h^-1^, which is smaller than the maximum specific growth rate observed on sorbitol. After a steady state (biomass around 24.6 g DCW/L) was reached during the continuous culture with sorbitol as sole carbon source, two consecutive transient continuous cultures were performed. The first one involved an increase in the feeding mixture of the methanol fraction from 0 to 1 and the second a decrease of the methanol fraction from 1 to 0.

**Figure 1:**
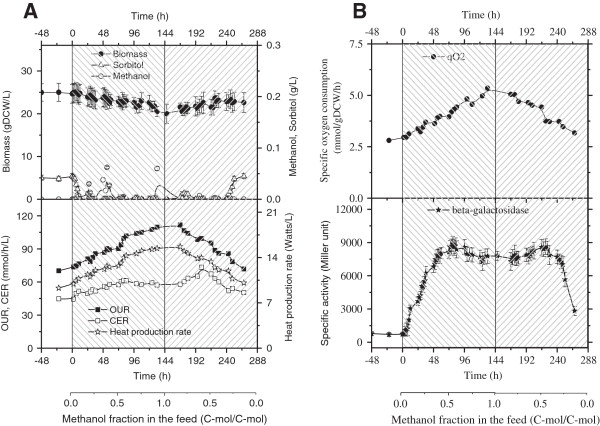
**Culture profiles during the two transient continuous cultures, as illustrated in the two shadowed blocks. (A)** Time courses of biomass, substrates, OUR, CER, and heat production; **(B)** time courses of specific oxygen consumption pO2 and β-galactosidase activities.

Figure 1A summarizes time courses of biomass, substrates, oxygen consumption (qO_2_) and heat production during the two transient continuous cultures. With methanol and sorbitol concentrations below 0.1 g/L, these cultures could be considered at dual substrate limitations, i.e., the two substrates were consumed simultaneously and almost completely. The biomass concentration was found to slightly decrease with the increase of methanol fraction, indicating a somewhat smaller biomass yield on methanol compared to sorbitol. By contrast, OUR and heat production increased nearly linearly with the increase of methanol fraction in the feeding mixture, and *vice versa*. Beside this, the qO_2_ increased from 2.88 mmol/(g DCW h) in absence of methanol to 4.41 mmol/(g DCW h) for 100% methanol (Figure 1B). Correspondingly, the heat production rate increased from 10.8 Watts/L to 16.5 Watts/L. Induction level of pAOX1, quantitatively estimated by means of β-galactosidase activities, was in a maximal range (7800 to 8600 Miller unit) when the methanol fraction was in the range of 0.45 ~ 0.75 C-mol/C-mol. Interestingly, β-galactosidase activity was increased slightly for the above mentioned optimal methanol/sorbitol ratio compared to that of 100% methanol. This highlighted that an appropriate methanol/sorbitol mixture could increase the pAOX1 induction level together with a reduction of oxygen consumption.

**Figure 2:**
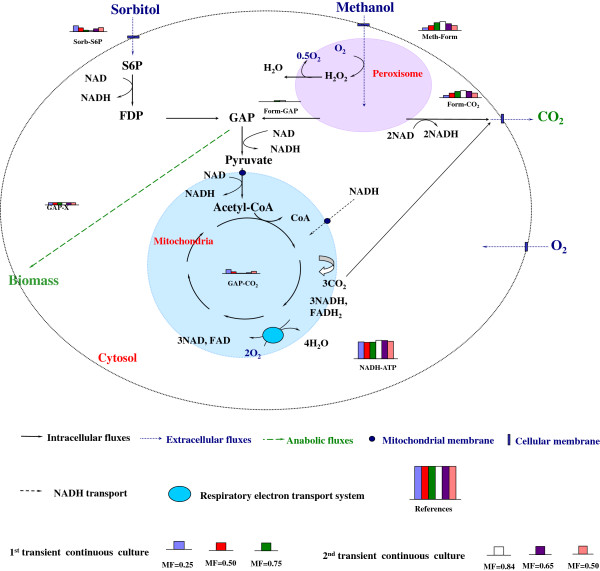
**Metabolic flux analysis at distinct physiological states corresponding to different methanol fractions in the methanol/sorbitol feed.** All fluxes have been normalized into the unit of C mmol/(g DCW · h) except the flux of *f*_*NADH-ATP*_ of which the unit is mmol/(g DCW · h). The scales of histograms of reference fluxes (i.e., references in Figure 2) are 10 C mmol/(g DCW · h) or 10 NADH mmol/(g DCW · h). Each flux is represented by the relative height of histogram.

In order to further understand the cooperation of both substrates, a MFA was performed based on a simplified model (Additional file 1: Table S1, Additional file 2), a cell chemical formula of *CH*_1.761_*O*_0.636_*N*_0.143_[23] and with the approximation that changes of biomass composition during all cultures could be neglected. The maximum likelihood metabolic distributions (a group of distinct metabolic states) were obtained for a series of methanol fraction values (Figure 2) after macroscopic data consistency check passed and reconciled extracellular rates were calculated (Additional file 1: Tables S2 and S3). Herein the aim of MFA is to answer how the dual carbon sources are used and cooperate during the co-feeding process rather than analyze the cellular metabolic network in detail.

At the first step of methanol utilization inside the peroxisome, methanol is oxidized into formaldehyde and hydrogen peroxide by alcohol oxidases (AOX, EC 1.1.3.13). The toxic byproduct H_2_O_2_ is then broken down into oxygen and water by specific catalase (CAT, EC 1.11.1.6). From the standpoint of energy generation, two reduction equivalents from oxydation *f*_*Meth-Form*_ (Additional file 1: Table S1) are wasted without ATP production. Histograms on Figure 2 show that the *f*_*Meth-Form*_ decreased with the decrease of methanol fraction in the feeding mixture while the energy production was complemented by the increase of sorbitol uptake flux *f*_*Sorb-S6P*_. As a result, almost the same level of ATP was produced with lower oxygen consumption. More precisely, the flux rate through the respiratory chain (*f*_*NADH-ATP*_) remained nearly constant around 5.2 ± 0.4 mmol/g DCW/h for the different methanol fractions considered. This indicated that Y_ATP/X_ was relatively constant during the cultures since the biomass specific growth rate was almost fixed (i.e., *f*_*GAP-X*_). Formaldehyde produced from methanol is subsequently oxidized either by sequent dehydrogenation reactions (represented by *f*_*Form-CO2*_) or condensed through a series of steps (represented by *f*_*Form-GAP*_) into glyceraldehyde 3-phosphate (GAP) by relevant enzymes. However, it was very difficult to exactly quantify the contribution of each substrate to catabolism (for energy generation, i.e., *f*_*GAP-CO2*_) and to anabolism (for biomass production, i.e., *f*_*GAP-X*_) due to the simplicity of the model used. Nevertheless, according to mass balance at the node of GAP, the minimal contribution from sorbitol to *f*_*GAP-X*_ can be obtained by *f*_*GAP-X*_*- f*_*Form-GAP*_. By this means, more than 61% of sorbitol was found dedicated to biomass production for methanol fraction of 0.5 C-mol/C-mol. This represented a value higher than 0.897 C mmol/g DCW/h of the total uptake rate (1.46 C mmol/(g DCW · h)) flowed through *f*_*GAP-X*_. At higher sorbitol proportions in the mixed feed, the consumed sorbitol was not only used for biomass production but also for ATP generation through *f*_*GAP-CO2*_ and *f*_*NADH-ATP*_. More precisely, the consumed sorbitol through TCA cycle was less than 39% at the methanol fraction of 0.5 C-mol/C-mol but more than 60% at the 0.25 C-mol/C-mol, respectively. Please refer to Additional file 2 for details.

To date, most studies in the field were focused mainly on the regulation of methanol utilization pathway at the gene level [24-26] and the influence of sorbitol on pAOX1 induction level has been seldom investigated [25]. In this work, based on a very simple metabolic model, MFA promoted a better understanding of the intracellular regulation processes of methanol/sorbitol co-feeding at the level of metabolism. Co-feeding of sorbitol can reduce the oxidation flux in the peroxisome, leading to less oxygen consumption and heat production. At the same time, sorbitol in the mixture produces energy through the TCA cycle and provides carbon source for biomass synthesis. According to the MFA model, it was found that 61% of sorbitol went through the TCA cycle and the rest was used for biosynthesis at 0.5 C-mol/C-mol methanol.

### Validation with fed-batch cultures

Fed-batch cultures were performed to further confirm results from the transient continuous cultures. Two types of feeding mixture were used: the first one with methanol as sole carbon source and the second one with a sorbitol/methanol mixture (methanol fraction 0.60 C-mol/C-mol).

**Figure 3:**
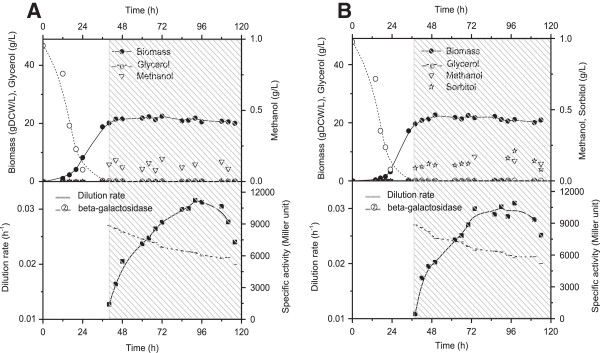
**Culture profiles during the control fed-batch culture with methanol as sole carbon source, as shown by the shadowed zone. (A)** Time courses of biomass, substrates, dilution rate, and β-galactosidase activities; **(B)**Time courses of biomass, substrates, dilution rate, and β-galactosidase activities during the control fed-batch culture with mixed feed (methanol fraction 0.60 C-mol/C-mol), as shown by the shadowed zone.

The culture volume increased from 1.5 L to around 2.0 L at the end of culture (due to regular sampling). As a result, the dilution factor was in a narrow range of 0.02 ~ 0.03 h^-1^. As shown in Figure 3, similar levels of biomass (20 ~ 23 g DCW/L) were obtained in both experimental conditions. This could be related to the comparable value of biomass yields (Y_X/S_, g DCW/C-mol) for methanol and sorbitol or to the equivalent total carbon concentration in both experiments (3.125 C-mol/L, i.e. the same as that in the transient continuous cultures). During the feeding processes, methanol and sorbitol concentrations were less than 0.25 g/L and the dilution rates decreased slowly from 0.027 h^-1^ to 0.020 h^-1^ at the end of cultures. For the control fed-batch culture (i.e. 100% methanol feeding), a maximal β-galactosidase activity (9000 ~ 11000 Miller unit) was observed from 84 h to 108 h, before decreasing then after. A similar level of β-galactosidase activities was obtained from 72 h to 108 h for the fed-batch culture with methanol/sorbitol co-feeding, demonstrating that the same induction level could be achieved with mixed carbon sources. These results confirm that optimally mixed methanol and sorbitol can provide comparable induction level of pAOX1 compared to methanol alone.

## Conclusions

In the present quantitative study on methanol/sorbitol co-feeding, it has been demonstrated clearly that an optimal mixture of methanol/sorbitol permits not only to reduce cell specific oxygen uptake, and thus heat production but also to maintain, even to increase, the level of pAOX1 induction. Therefore, the advantages of methanol/sorbitol co-feeding are multifold (as shown in Table 1). Actually, further experiments are in progress to determine the optimal feeding rates in order to maximize productivities, such as biomass and protein of interest.

**Table 1:** Highlighted benefits of methanol/sorbitol co-feeding, T = 25°C, and D = 0.023 h^-1^

	**Methanol**	**Methanol/sorbitol 0.50/0.50 C-mol/C-mol**	**Unit**
Specific oxygen consumption	4.8 ± 0.5	3.7 ± 0.4	mmol/(g DCW · h)
Specific heat production	0.67 ± 0.07	0.51 ± 0.06	Watts/g DCW
Specific activity of β-galactosidase	7.8 ± 0.7	8.6 ± 0.8	×10^3^ Miller unit
Theoretical maximum biomass	1.1 ~ 1.4*	1.5 ~ 1.8*	×10^2^ g DCW

* Assume maximum OTR = 600 mmol/(L · h).

## Methods

### Strain and medium composition

A *P. pastoris* strain GS115 transformed with pSAOH5 vector bearing a pAOX1-LacZ construct was used [27,28]. Media used were named as follows: BG (batch culture with glycerol); CS (1^st^ continuous culture with sorbitol), TCC (2^nd^ transient continuous culture with methanol/sorbitol co-feeding), FBM (fed-batch with methanol), and FBMS (fed-batch with methanol/sorbitol mixture). Medium compositions was as follows (per liter) : 25 mL H_3_PO_4_ 85%, 1.05 g CaSO_4_.2H_2_O, 18.28 g K_2_SO_4_, 14.96 g MgSO_4_.7H_2_O, 4.15 g KOH, and 12 mL PTM1 trace elements solution (*P. pastoris* fermentation manual of the Invitrogen) and 3.2 C-mol/L of the carbon source. These were 0.54 mol/L glycerol, 3.2 C-mol/L sorbitol, 3.2 C-mol/L methanol/sorbitol mixture (linear change of the methanol fraction), C-mol/L methanol, and 3.2 C-mol/L methanol/sorbitol mixture (0.60/0.40 C-mol/C-mol), in BG, CS, TCC, FBM, and FBMS medium, respectively.

### Bioreactor operation

All cultures were performed in 2 l bioreactor (Biostat® B plus, Sartorius AG). The temperature was maintained at 30°C during glycerol growth phase and shifted to 25°C during methanol and mixed feed induction phase. The pH was regulated at 5.8±0.2 by the addition of 25% ammonia solution. Dissolved oxygen (DO) was maintained at 30% of saturation by a PID controller . The culture history was recorded by the supervisory control and data acquisition system (MFCS/win 3.0).

Culture started in batch mode with 1.5 l of the BG medium at an initial OD600 of 0.2. After glycerol depletion (observed typically after 32 ~ 36 h with an sudden increase of pO_2_), a continuous culture phase started by feeding the bioreactor with the CS medium at a dilution rate of 0.023 h^-1^ (corresponding to a feeding rate of 46 ml/h). After a steady state was reached (i.e. after 5 residence times), the transient continuous cultures were carried out by increasing (1^st^ transient continuous culture) or decreasing (2^nd^ transient continuous culture) the methanol fraction in the TCC medium. The dilution rate was also maintained at 0.023 h^-1^ during the transient continuous cultures.

Fed-batch cultures started with a batch phase in 1.5 L BG medium at an initial OD600 of 0.2. After glycerol depletion, the cultures were fed with the FBM medium or FBMS medium. The feeding rate was fixed at 40 ml/h for two fed-batch cultures and the dilution rate was calculated based on the increase of the culture volume over time.

### Quantitative analysis of biomass, metabolites, and β-galactosidase

Cell growth was monitored either by optical density at 600 nm (OD600) or dry cell weight (DCW). An OD600 value of 1 was found to correspond to 0.236 g DCW/L. Glycerol, methanol, and sorbitol were analyzed by isocratic RID-HPLC (Hewlett Packard model 1100, Waldbronn, Germany) using an Aminex HPX-87H ion-exclusion column (300×7.8 mm Bio-Rad, Hercules, USA) with 5 mmol/L H_2_SO_4_ as mobile phase at a flow rate of 0.5 ml/min at 30°C. Ammonia assay is based on the phenol-hypochlorite method [29]. β-galactosidase activities were determined as described previously [30]. All analyses were performed in duplicate. Statistical significance was accepted at *p* > 0.05.

### Exhaust gas analysis and heat production calculation

The concentrations of oxygen and carbon dioxide in the exhaust gas were monitored in real time with a gas analyzer (EGAS-1, Advance optima, ABB). OUR (CER) were assumed to equal OTR (CTR) and were calculated by below equations:

(1) $$\text{OUR}≅\text{OTR}=1000\times \left(\frac{O_{2}{}^{\text{in}}}{100}-\frac{N_{2}{}^{\text{in}}\times O_{2}{}^{\text{out}}}{100\times N_{2}{}^{\text{out}}}\right)\times \frac{Q_{g}}{V}\times \frac{60}{V_{m}}$$

(2) $$\text{CER}≅\text{CTR}=1000\times \left(\frac{N_{2}{}^{\text{in}}\times CO_{2}{}^{\text{out}}}{100\times N_{2}{}^{\text{out}}}-\frac{CO_{2}{}^{\text{in}}}{100}\right)\times \frac{Q_{g}}{V}\times \frac{60}{V_{m}}$$

Heat production was calculated by the following equation [8]:

(3) $$\begin{array}{ll} –\text{Δ}H_{\text{reaction}} & ≅–\text{Δ}H^{o}{}_{\text{reaction}}\\ & ≅\left[{\left(\sum_{j}Y_{s,j}{\text{κ}}_{j}\right)}_{\text{substrates}}-{\left(\sum_{i}Y_{p,i}{\text{κ}}_{i}\right)}_{\text{products}}\right]\\ & \times 115\;\text{KJ}/C-\text{mol} \end{array}$$

### Extracellular flux ***f***_*extra*_ determination

Totally extracellular fluxes of 5 species from the measurements during transient cultures could be determined as follows.

The specific biomass growth rate (μ) was determined by:

(4) $$V\frac{\mathrm{dX}}{\mathrm{dt}}=\mathrm{\mu XV}–\mathrm{FX}\;≅\;0\;\Rightarrow \;\mu \;≅\;\frac{F}{V}=D$$

The specific production rate of biomass *f*_*extra*,*X*_ was obtained in the unit of mmol/(g DCW· h):

(5) $$f_{\mathrm{extra},X}=1000\times \frac{\mu }{M_{X}}$$

A standard chemical composition CH_1.761_O_0.636_N_0.143_[23] was used, and the ash content of biomass was assumed to be 5%. As a result, M_X_ of 31 g DCW/mol was obtained.

The specific consumption of oxygen was obtained after OUR measurement:

(6) $$f_{\mathrm{extra},O_{2}}=–\frac{\text{OUR}}{X}$$

The specific production of carbon dioxide was calculated by:

(7) $$f_{\mathrm{extra},CO_{2}}=\frac{\text{CER}}{X}$$

The specific rates of substrate consumption were determined from the following equation:

(8) $$\begin{array}{ll} V\frac{dC_{i}}{\text{dt}} & =f_{\mathrm{extra},C_{i}}\text{XV}+F\left(C_{i,\mathrm{in}}–C_{i}\right)=0\Rightarrow f_{\mathrm{extra},C_{i}}\\ & =–\frac{D\left(C_{i,\mathrm{in}}–C_{i}\right)}{X}\\ i & =\text{methanol},\;\text{sorbtiol} \end{array}$$

### Data reconciliation and consistency check of macroscopic balances

The macroscopic balances of carbon element (C) and redox degree provided two degrees of redundancy Additional file 2. The reconciled measurement vector ***f*** *_extra_ was the minimum variance estimate of measurement noise (error) which was assumed to distribute independently and normally with a mean value of zero:

(9) $$f_{\mathrm{extra}}^{*}=\mathrm{Arg}\;\min_{f*\mathrm{extra}}{\left(f_{\mathrm{extra}}^{*}–f_{\mathrm{extra}}\right)}^{T}W\left(f_{\mathrm{extra}}^{*}–f_{\mathrm{extra}}\right)$$

s.t.

(10) $$R_{\mathrm{redundancy}}f_{\mathrm{extra}}^{*}=0$$

where ***R***_*redundancy*_ ∈ ***R***^2 × 5^ was the redundancy matrix of which each element of the first or second row was the C number or redox degree of one species molecule; ***f***_*extra*_ ∈ ***R***^5 × 1^ was the measured extracellular flux vector. ***W*** was the diagonal weighting matrix of which each element w_i,i_ equals 1/*σ*_*i*_^2^ (i.e., the reciprocal of squared standard deviation of *f*_*extra*,*i*_ ). In this work, *σ*_*i*_ was assumed to be a constant percent of *f*_*extra*,*i*_. The error percentage was 10% for biomass, biomass, methanol, and sorbitol measurements, and 15% for OUR and CER measurements.

The solution of Eq. (9) was obtained by the following equations [31]:

(11) $$f_{\mathrm{extra}}^{*}=\left(I–W^{–1}R_{\mathrm{redundancy}}{}^{T}P_{\varepsilon }{}^{–1}R_{\mathrm{redundancy}}\right)f_{\mathrm{extra}}$$

(12) $$P_{\varepsilon }=R_{\mathrm{redundancy}}W^{-1}R_{\mathrm{redundancy}}{}^{T}$$

Additionally, a statistical consistency index *h*_ε_, following a χ–square distribution was used to check the consistency of macroscopic balance constraints [31].

(13) $$h_{\varepsilon }=\varepsilon^{T}P_{\varepsilon }{}^{–1}\varepsilon$$

where ***ε*** = ***R***_*redundancy*_ ***f***_*extra*_ was the vector of balance residual. All calculations were performed in the platform of Matlab 2011b (http://www.mathworks.com).

### Intracellular metabolic flux *f*_*intra*_ analysis

Additional file 1: Table S4 in Supplementary Material 1 shows the details of proposed metabolic model. Firstly, NADH and NADPH were considered to be equivalent or transform into each other liberally, so NADH was only used to represent both of them. Secondly, summation of a group of sequential elementary metabolic reactions without branched pathway led to an equivalency of overall reaction (such as *f*_*GAP-CO2*_). Finally, production of biomass was simply represented by an overall reaction, into which relevant complicated anabolic reactions from the precursor GAP were lumped macroscopically based on carbon and reduction balance.

Intracellular flux ${\hat{f}}_{\mathrm{intra}}$ was estimated by solving a constrained linear system below

(14) $$\begin{array}{l} R_{\mathrm{extra}}\cdot {\hat{f}}_{\mathrm{intra}}=f_{\mathrm{extra}}^{*}\\ R_{\mathrm{intra}}\cdot {\hat{f}}_{\mathrm{intra}}=0\\ \text{s}.\text{t}.\;{\hat{f}}_{\mathrm{intra}}\geq 0 \end{array}$$

The first two equations in Eq. (14) were mass balances for extracellular, and intracellular species (due to pseudo steady-state assumption and neglecting dilution effect of cell growth), respectively. And the third corresponds to the constraint of thermodynamic irreversibility. ***R***_*extra*_ ∈ ***R***^5 × 10^ and ***R***_*intra*_ ∈ ***R***^6 × 10^ were corresponding stoichimetric matrices (Additional file 2). As a whole, the proposed metabolic model has ten reactions needing estimation while five extracellular rates were measurable and six intermediates were assumed to be steady-state. As a result, the proposed model is over-determined.

Additionally, sensitivity and singularity test [31] were performed to ensure that the stoichiometric system was well-posed (i.e., the stoichiometric matrix was well-conditioned).

## Abbreviations

C-mol: Carbon molar; S6P: Sorbitol-6-phosphate; FDP: Fructose-1,6-diphosphate; GAP: Glyceraldehyde-3-phosphate; Ci: Substrate concentration (mmol/L); F: Feeding rate (L/h); D: Dilution rate (h^-1^); ΔHoreaction: Molar standard entropy of reaction (KJ/C-mol); MX: The molecular weight of biomass (31 g DCW/mol); Qg: Air flow rate (slpm/min); V: Culture volume in the bioreactor (l); Vm: Standard molar volume of the gas (22.414 l/mol); X: Scalar of total, active, inactive biomass in bioreactor (g DCW/L); YS,j: Y_P,i_, Coefficient (yield) of substrate, production to biomass produciton(C-mol/C-mol); CER: CTR, Carbon dioxide evolution rate, transfer rate (mmol/(L h)); CO2in: CO_2_^out^, Carbon dioxide in the inflow, outflow of exhaust gas (% v/v); MFA: Metabolic flux analysis; N2in: N_2_^out^, Nitrogen in the inflow, outflow of exhaust gas (% v/v); OTR: OUR, Oxygen transfer rate, uptake rate (mmol/(L h)); P/O: Atoms of phosphate incorporated as ATP per atom oxygen; yATP/X: ATP coefficient for biomass synthesis (mmol/mmol); κ: Reduction degree; μ: Cell specific growth rate (h^-1^).

## Competing interests

The authors declare that they have no competing interests.

## Authors’ contributions

Conceived and designed the experiments: HN, PF. Performed cultures in bioreactor: HN, HS. Constructed the Mut+/pSAOH5 strain; NP, HN. Performed HPLC analysis: LJ. Analyzed the data and wrote the paper: HN, PF, MD, CR. All authors read and approved the final manuscript.

## Supplementary Material

Additional file 1: Table S1 The simplified metabolic reactions used for MFA in this study. **Table S2**. Measured *f*_*exta*,*i *_and reconciled *f*^∗^_*extra*,*i *_specific rates (mmol/(g DCW h)) of metabolites during the first transient continuous culture with the change of methanol fraction (C-mol/C-mol). **Table S3**. Measured *f*_*exta*,*i *_and reconciled *f*^∗^_*extra*,*i *_specific rates (mmol/(g DCW h)) of metabolites during the second transient continuous culture with the change of methanol fraction (C-mol/C-mol). Click here for file

Additional file 2 The excel file for MFA, including the sheet of reconciled extracellular specific rates, the sheet of stoichiometric matrix, the sheet of MFA data, and the sheet of visualized MFA. Click here for file
