# Supplementary material for: A quantitative study of methanol/sorbitol co-feeding process of a Pichia pastoris Mut+/pAOX1-lacZ strain
Source: Microb Cell Fact. 2013 Apr 8;12:33. doi: 10.1186/1475-2859-12-33 (PMC3639866; doi:10.1186/1475-2859-12-33)
Supplement: Additional file 1: Table S1 — The simplified metabolic reactions used for MFA in this study. Table S2. Measured fexta,i and reconciled f∗extra,i specific rates (mmol/(g DCW h)) of metabolites during the first transient continuous culture with the change of methanol fraction (C-mol/C-mol). Table S3. Measured fexta,i and reconciled f∗extra,i specific rates (mmol/(g DCW h)) of metabolites during the second transient continuous culture with the change of methanol fraction (C-mol/C-mol). [file 1475-2859-12-33-S1.doc]

Table S1. The simplified metabolic reactions used for MFA in this study

| Pathway | Reaction |
| --- | --- |
| *f_Sorb-S6P_* | Sorbitol +ATP → Sorbitol-6-phosphate + ADP |
| *f_S6P-FDP_* | Sorbitol-6-phosphate + ATP + NAD → Fructose-1,6-phosphate + ADP + NADH |
| *f_FDP-GAP_* | Fructose-1,6-phosphate → 2 Glyceraldehyde-3-phosphate |
| *f_Meth-Form_* | Methanol + (1/2) × O_2_→ Formaldehyde + H_2_O |
| *f_Form-CO2_* | Formaldehyde + 2 NAD → CO_2_ + 2 NADH |
| *f_Form-GAP_* | Formaldehyde + ATP → (1/3) × Glyceraldehyde-3-phosphate + ADP |
| *f_GAP-CO2_* | Glyceraldehyde-3-phosphate + 5 NAD + FAD + 2 ADP + GDP →  3 CO_2_ + 5 NADH + FADH_2_ + 2 ATP + GTP |
| *f_NADH-ATP_* | NADH + (1/2) × O_2_ + (P/O) × ADP → NAD + (P/O) × ATP + H_2_O |
| *f_FADH2-NADH_* | FADH_2_ + GTP + NAD → NADH + GDP + FAD |
| *f_GAP-X_* | (1/3) × Glyceraldehyde-3-phosphate + (1/2)×(14/3-4.06) × NADH + Y_ATP/X_ × ATP →  X(CH_α_N_β_O_γ_) ^*^ + (1/2)×(14/3-4.06) × NAD + Y_ATP/X_ × ADP |

*: biomass in chemical formula CH_α_N_β_O_γ_ ;

Table S2. Measured ($f_{exta,i}$) and reconciled ($f^{*}_{extra,i}$) specific rates (mmol/(g DCWh)) of metabolites during the first transient continuous culture with the change of methanol fraction (C-mol/C-mol).

| Methanol fraction | 0 | | 0.25 | | 0.50 | | 0.75 | |
| --- | --- | --- | --- | --- | --- | --- | --- | --- |
|  | $f_{exta,i}$ | $f^{*}_{extra,i}$ | $f_{exta,i}$ | $f^{*}_{extra,i}$ | $f_{exta,i}$ | $f^{*}_{extra,i}$ | $f_{exta,i}$ | $f^{*}_{extra,i}$ |
| Methanol | 0 | 0 | -0.87 | -0.80 | -1.64 | -1.70 | -2.86 | -2.71 |
| Sorbitol | -0.55 | -0.49 | -0.38 | -0.40 | -0.25 | -0.26 | -0.11 | -0.11 |
| X | 0.92 | 0.95 | 0.92 | 0.91 | 0.92 | 0.91 | 0.92 | 0.94 |
| O_2_ | -2.88 | -2.20 | -3.57 | -2.85 | -3.83 | -3.27 | -4.03 | -3.81 |
| CO_2_ | 1.57 | 1.98 | 2.19 | 2.27 | 2.26 | 2.32 | 2.51 | 2.43 |
| *h_ε_* | 6.88 | | 1.69 | | 1.16 | | 1.25 | |
| *χ^2^* | 5.99 | | 5.99 | | 5.99 | | 5.99 | |
| Pass/fail | Fail | | Pass | | Pass | | Pass | |

Table S3. Measured ($f_{exta,i}$) and reconciled ($f^{*}_{extra,i}$) specific rates (mmol/(g DCWh)) of metabolites during the second transient continuous culture with the change of methanol fraction (C-mol/C-mol).

| Methanol fraction | 0.84 | | 0.65 | | 0.50 | | 0.27 | |
| --- | --- | --- | --- | --- | --- | --- | --- | --- |
|  | $f_{exta,i}$ | $f^{*}_{extra,i}$ | $f_{exta,i}$ | $f^{*}_{extra,i}$ | $f_{exta,i}$ | $f^{*}_{extra,i}$ | $f_{exta,i}$ | $f^{*}_{extra,i}$ |
| Methanol | -2.94 | -3.09 | -2.18 | -2.35 | -1.59 | -1.71 | -0.78 | -0.84 |
| Sorbitol | -0.09 | -0.09 | -0.19 | -0.20 | -0.26 | -0.28 | -0.29 | -0.33 |
| X | 0.92 | 0.91 | 0.92 | 0.89 | 0.92 | 0.89 | 0.92 | 0.86 |
| O_2_ | -4.92 | -4.29 | -4.49 | -3.89 | -4.18 | -3.45 | -3.13 | -2.49 |
| CO_2_ | 2.71 | 2.72 | 2.96 | 2.64 | 2.84 | 2.53 | 2.03 | 1.93 |
| *h_ε_* | 1.06 | | 2.15 | | 3.15 | | 6.82 | |
| *χ^2^* | 5.99 | | 5.99 | | 5.99 | | 5.99 | |
| Pass/fail | Pass | | Pass | | Pass | | Fail | |
